# Dedifferentiated Liposarcoma: A Rare Case Report of Retroperitoneal Myxoid Soft Tissue Tumour with Diagnostic Dilemma

**DOI:** 10.1177/2632010X221112455

**Published:** 2022-07-19

**Authors:** Ritica Chaudhary, Mona Lisa, Payal Kumari, Aman Kumar

**Affiliations:** 1 Department of Pathology, IGIMS, Patna, Bihar, India; 2 Department of Surgery, UNC School of Medicine, Chapel Hill, NC, USA

**Keywords:** Dedifferentiated liposarcoma, MDM2, CDK4, retroperitoneum, IHC, FISH

## Abstract

**Background:**

The retroperitoneum can host a wide spectrum of soft tissue lesions. These tumours pose a challenge to the pathologist as the morphology is not of much help and immunohistochemistry becomes a necessity.

**Case report:**

Sixty years old male presented with 2 months history of abdominal lump, pain and dyspepsia. The MRI revealed a heterogeneous mass in the retroperitoneum involving right para spinal muscle, right iliac fossa and right perinephric region with destruction of right transverse process and erosion of adjacent L3 vertebra. Trucut biopsy of the mass was reported as fibroliposarcoma at an outside lab. Patient underwent a wide local excision. Grossly the tumour gave an impression of a liposarcoma but the microscopy showed areas of spindle cells, epitheloid cells, focal areas of ganglion like cells and large areas of myxoid change. IHC panel of S-100, SMA, caldesmon, myogenin, myoglobin and Alk-1 was negative. MDM2, CDK4 and p16 IHC came positive proving it to be a dedifferentiated liposarcoma.

**Conclusion:**

We report a curious case of retroperitoneal soft tissue tumour with complex morphology and IHC features diagnosed as dedifferentiated liposarcoma based on MDM2, CDK and p16 positivity.

## Introduction

The retroperitoneum can host a wide spectrum of soft tissue lesions. Among these, liposarcoma is the most common soft tissue sarcoma. WHO classification categorizes liposarcoma into 5 categories, out of which well differentiated liposarcoma constitutes around 40%.^1,2^ About 10% of these tumours dedifferentiate and make the diagnosis difficult.^2^ These tumours pose a challenge to the pathologist as the morphology is not of much help and immunohistochemistry becomes a necessity. One of the characteristic as well as distinguishing feature of dedifferentiated liposarcoma is that it is usually located in the retroperitoneum unlike well differentiated liposarcoma, which is most often seen in limbs. On microscopy, dedifferentiated liposarcoma shows areas of atypical lipomatous tumours but most of the areas show dedifferentiated neoplastic tissue. This dedifferentiated tumour can make this tissue look like complex neoplasms such as malignant fibrous histiocytoma, chondrosarcoma, osteosarcoma, angiosarcoma or sometimes even as melanoma, meningioma, lymphoma.^3-7^ Even though it appears that dedifferentiated liposarcoma would develop from well differentiated liposarcoma, many cases are found de novo as well.^8^ Dedifferentiation is a process of progression from well differentiated form to a higher-grade less differentiated form. In most circumstances dedifferentiation worsens the prognosis. However, in cases of dedifferentiated liposarcoma, neither the local extent nor the grade has any significant influence on the behaviour or prognosis of this tumour.^6-8^ We report such a case of a retroperitoneal mass diagnosed as fibroliposarcoma on biopsy gave a completely different histological picture on final histopathology. It required a whole set of IHC and FISH to establish the diagnosis.

## Case Report

**Figure 1:**
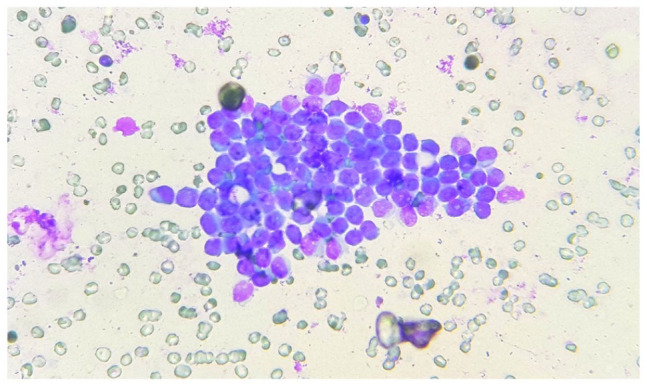
FNAC: low power (10× view) clusters of small round cells with scant cytoplasm.

A sixty years old male presented with 2 months history of abdominal lump, pain and dyspepsia. On clinical examination around 10 cm × 10 cm non-tender, retroperitoneal tumour was palpable. MRI of the abdomen revealed a 15 cm × 12 cm heterogeneous retroperitoneal mass involving right para spinal muscle extending deep into reteroperitoneum, right iliac fossa and right perinephric region with destruction of right transverse process and erosion of adjacent L3 vertebra. FNAC of the mass was performed and it showed features of fibroliposarcoma (Figure 1). Patient was investigated further and found to have a negative metastatic workup. He underwent a wide local excision of the mass with surrounding tissue including muscles and a part of bone. Margins were labelled and sent for a frozen section which revealed negative margins. The excised tumour with labelled margins was sent for final histopathological examination.

**Figure 2.**
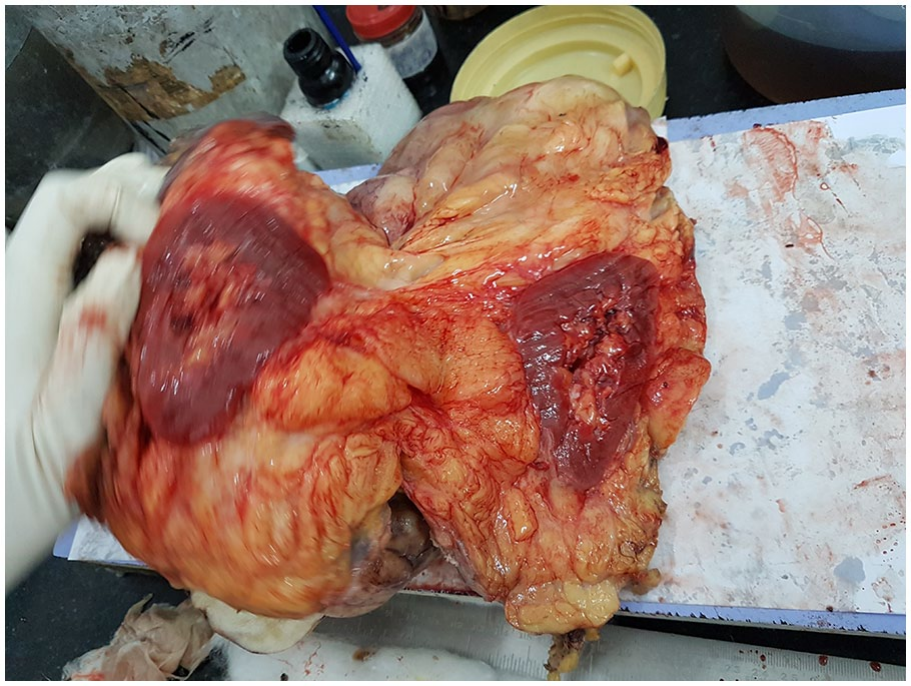
Gross morphology: Fatty tumour encasing the right kidney.

**Figure 3.**
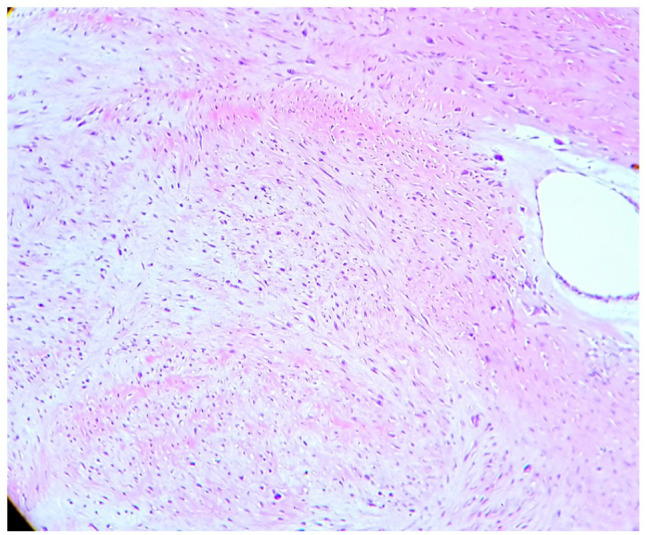
H & E Staining: low power (10× view) areas of tumour showing spindle cells and epitheloid cells with occasional scattered cells having large hyperchromatic nuclei and scant cytoplasm.

**Figure 4.**
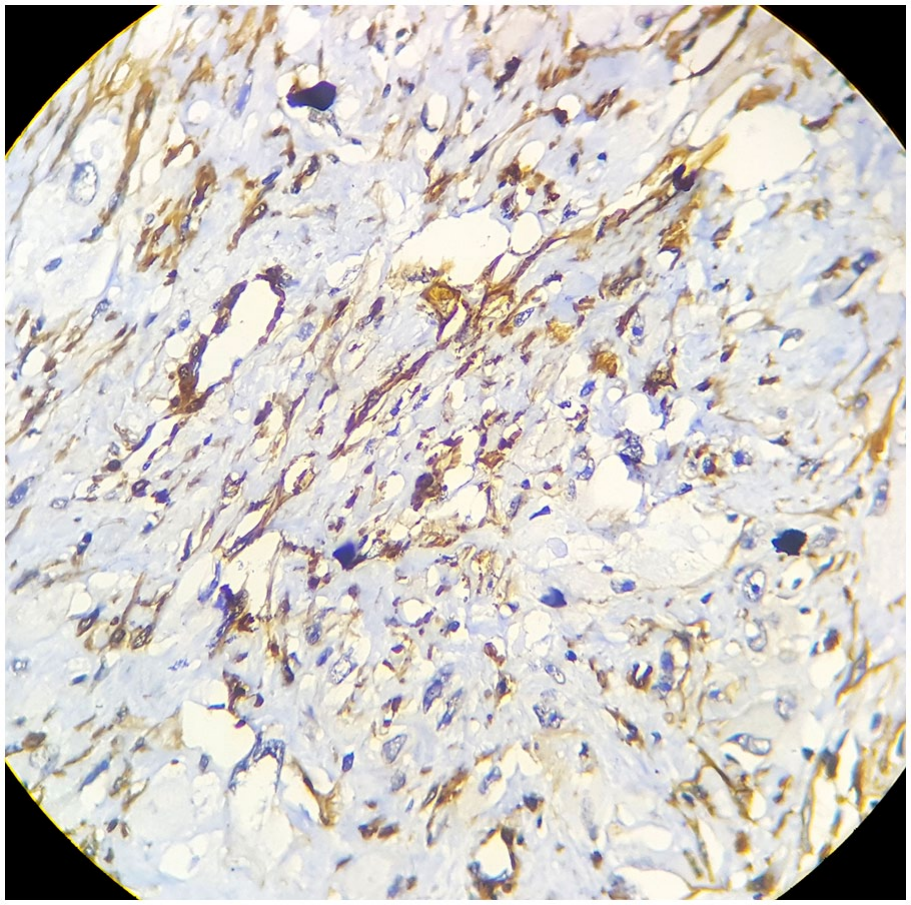
SMA Negative: high power (40× view) tumour cells are SMA negative, blood vessels and connective tissues acting as positive control.

**Figure 5.**
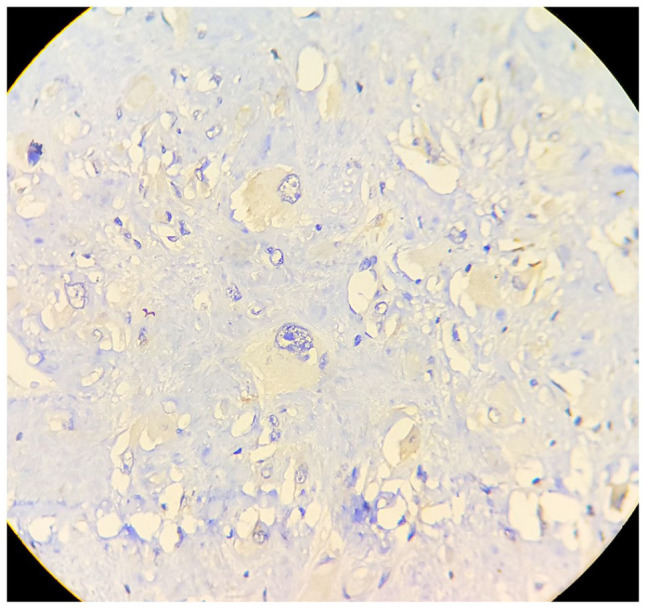
S 100 Negative: high power (40× view) tumour cells are S100 negative.

**Figure 6.**
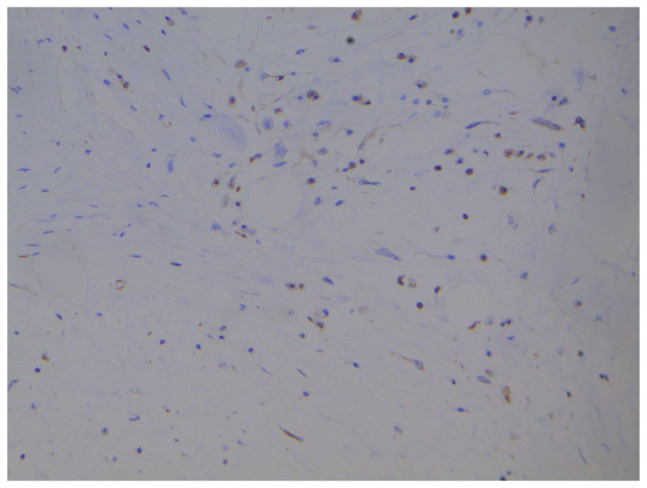
MDM2 : high power (40× view) strong nuclear positivity seen in tumour cells.

**Figure 7.**
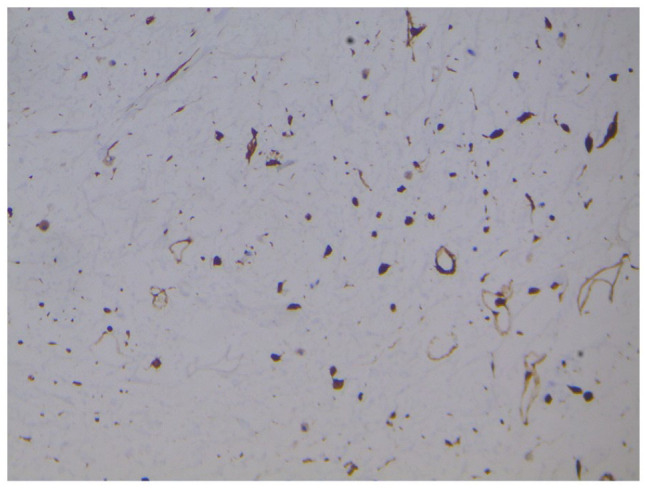
CDK4: high power (40× view) strong nuclear positivity seen in tumour cells.

**Figure 8.**
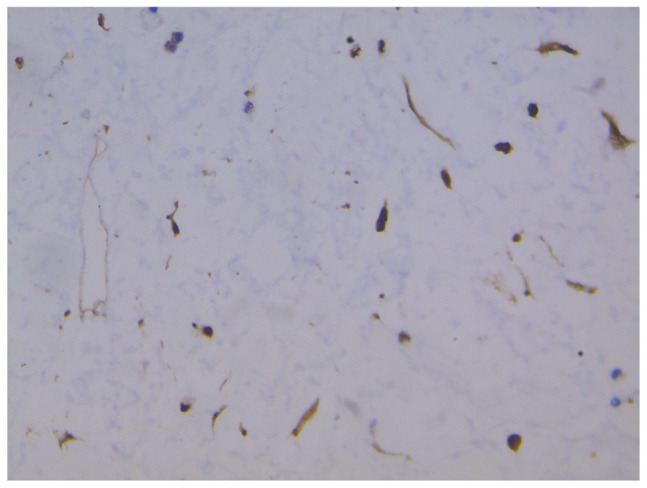
p16: high power (40× view) strong nuclear positivity seen in tumour cells.

On gross examination the 15 cm × 12 cm × 7 cm specimen contained a 7 cm × 3.5 cm × 3.1 cm tumour with whitish/pale yellow cut surfaces. Grossly the tumour gave an impression of a liposarcoma but the microscopy was altogether a different story with areas of spindle cells, epitheloid cells, focal areas of ganglion like cells and large areas of myxoid changes (Figure 2). Haematoxylin and eosin staining showed a neoplasm with high cellularity and mostly spindle-shaped cells arranged in storiform pattern (Figure 3). Looking at the histological picture, 2 differentials namely, myxoid liposarcoma and MPNST were kept in mind and an IHC panel of S-100, SMA, Caldesmon, myogenin, myoglobin and Alk-1 was applied (Figures 4 and 5). All of these IHC panels came negative and increased the complexity and dilemma of diagnosis. Subsequently other immunostainings such as MDM2, cdk4 and p16 were performed (Figures 6–8). It turned out to be positive for MDM2, cdk4 and p16 pointing the diagnosis towards dedifferentiated liposarcoma in the light of complex differentiated and dedifferentiated tissue on microscopy. FUS-DDIT3 was negative in the tumour on PCR.

## Discussion

Retroperitoneal tumour is an overall rare entity accounting for less than 0.2% of all malignant tumours. It is most commonly seen in males in their 4th to 5th decade of life. Around 40% of all the retroperitoneal tumours is liposarcoma.^2^ As per WHO classification, it has 5 subtypes namely, well differentiated, myxoid, round cell, pleomorphic and dedifferentiated types.^1^ Dedifferentiated liposarcoma has histological features of both well differentiated and poorly differentiated liposarcoma along with non-lipomatous areas. Mostly, this tumour is asymptomatic and identified incidentally on imaging as a part of routine medical examination. However, it can present with abdominal lump, pain or discomfort, or sometimes obstructive or compressive symptoms. CT scan, can be used to assess the extent and anatomy. Biopsy is usually performed before surgery and correlated with radiology to reach a diagnosis.

Even though most retroperitoneal tumours are diagnosed on microscopy using regular stains, sometimes they require special stains to rule out certain pathologies. These stains are SMS, S100, actin, desmin, caldesmon, myogenin, myoglobin, Alk-1, etc. Sometimes, IHC is required to get to a diagnosis and thus it increases the complexity of the case such as differentiated liposarcoma. Atypical lipomatous tumours and dedifferentiated liposcarcoma shows a characteristic genomic amplification of 12q13 to 15. We could not test the copy number changes in chromosome 12 as the test was not available at our centre. It includes the proto-oncogenes MDM2 and CDK4. So MDM2 and CDK4 staining plays a very important role in the diagnosis of dedifferentiated liposarcoma. Binh et al^9^ in their study described that more than 90% dedifferentiated liposarcoma showed a MDM2 and CDK4 staining showing that MDM2 and CDK4 immunohistochemistry is a sensitive technique in the diagnosis of dedifferentiated liposarcoma. MDM2 amplification has been described in malignant fibrous histiocytoma and other sarcomas as well such as MPNST, PNET, rhabdomyosarcoma, low grade or dedifferentiated osteosarcoma and synovial sarcoma.^10^ However, another study involving malignant fibrous histiocytoma have shown that some of these cases in the past might have been misclassified and were indeed dedifferentiated liposarcoma.^11^ Dedifferentiated liposarcoma has also been described to have overexpression of cell cycle regulator p16. Thway et al^12^ described the utility of immunohistochemistry for MDM2, CDK4 and p16 in the routine diagnosis of dedifferentiated liposarcoma. However, there have been conflicting studies showing lesser usefulness of p16 as compared to MDM2and CDK4.^13^ In our case, all the 3 IHC markers namely, MDM2, CDK4 and p16 were positive showing the usefulness of these markers in such complex cases with atypical features. Techniques like CGH, FISH and quantitative PCR can also be employed to further confirm the diagnosis.

## References

[1] Fletcher CDM . WHO: in classification of tumours of soft tissue and bone. In: Bridge JA Hogendoorn PCW Mertens F , eds. World Health Orgn. 4th ed. WHO PRESS; 2013:468.

[2] Mentzel T Schneider-Stock R . Lipogen differenzierte Tumoren. In: Klöppel G Kreipe HH Remmele W , eds. Pathologie (Kopf-Hals-Region, Weichgewebstumoren, Haut). 3rd ed. Springer; 2008:401-403.

[3] Nascimento AG. Dedifferentiated liposarcoma. Semin Diagn Pathol. 2001;18:263-266. 11757866

[bibr4-2632010X221112455] Evans HL Khurana KK Kemp BL Ayala AG. Heterologous elements in the dedifferentiated component of dedifferentiated liposarcoma. Am J Surg Pathol. 1994;18:1150-1157. 7943536 10.1097/00000478-199411000-00009

[bibr5-2632010X221112455] Nascimento AG Kurtin PJ Guillou L Fletcher CDM . Dedifferentiated liposarcoma: a report of nine cases with a peculiar neural like whorling pattern associated with metaplastic bone formation. Am J Surg Pathol. 1998;22:945-955. 9706974 10.1097/00000478-199808000-00004

[bibr6-2632010X221112455] Fanburg-Smith JC Miettinen M. Liposarcoma with meningothelial-like whorls: a study of 17 cases of a distinctive histological pattern associated with dedifferentiated liposarcoma. Histopathology. 1998;33:414-424. 9839165 10.1046/j.1365-2559.1998.00536.x

[bibr7-2632010X221112455] Hasegawa T Seki K Hasegawa F , et al. Dedifferentiated liposarcoma of retroperitoneum and mesentery: varied growth patterns and histological grades–a clinicopathologic study of 32 cases. Hum Pathol. 2000;31:717-727. 10872666 10.1053/hupa.2000.8222

[8] Henricks WH Chu YC Goldblum JR Weiss SW. Dedifferentiated liposarcoma: a clinicopathological analysis of 155 cases with a proposal for an expanded definition of dedifferentiation. Am J Surg Pathol. 1997;21:271-281. 9060596 10.1097/00000478-199703000-00002

[9] Binh MB Sastre-Garau X Guillou L , et al. MDM2 and CDK4 immunostainings are useful adjuncts in diagnosing well-differentiated and dedifferentiated liposarcoma subtypes: a comparative analysis of 559 soft tissue neoplasms with genetic data. Am J Surg Pathol. 2005;29:1340-1347. 16160477 10.1097/01.pas.0000170343.09562.39

[10] Momand J Jung D Wilczynski S Niland J. The MDM2 gene amplification database. Nucleic Acids Res. 1998;26:3453-3459. 9671804 10.1093/nar/26.15.3453 PMC147746

[11] Coindre JM Mariani O Chibon F , et al. Most malignant fibrous histiocytomas developed in the retroperitoneum are dedifferentiated liposarcomas: a review of 25 cases initially diagnosed as malignant fibrous histiocytoma. Mod Pathol. 2003;16:256-262. 12640106 10.1097/01.MP.0000056983.78547.77

[12] Thway K Flora R Shah C Olmos D Fisher C. Diagnostic utility of p16, CDK4, and MDM2 as an immunohistochemical panel in distinguishing well-differentiated and dedifferentiated liposarcomas from other adipocytic tumors. Am J Surg Pathol. 2012;36:462-469. 22301498 10.1097/PAS.0b013e3182417330

[13] Kang Y Horvai AE. P16 immunohistochemistry is less useful than MDM2 and CDK4 to distinguish dedifferentiated liposarcomas from other retroperitoneal mimics. Appl Immunohistochem Mol Morphol. 2017;25:58-63. 26509911 10.1097/PAI.0000000000000270

